# Suppression of Methicillin-Resistant Staphylococcus aureus and Reduction of Other Bacteria by Black Soldier Fly Larvae Reared on Potato Substrate

**DOI:** 10.1128/spectrum.02321-22

**Published:** 2022-10-05

**Authors:** Marissa Kinney, Matthew Moyet, Edward Bernard, Andrei Alyokhin

**Affiliations:** a Department of Molecular and Biomedical Sciences, University of Maine, Orono, Maine, USA; b School of Biology and Ecology, University of Maine, Orono, Maine, USA; The Pennsylvania State University

**Keywords:** black soldier flies, *Hermetia illucens*, methicillin-resistant *Staphylococcus aureus*, microbial contamination, waste management

## Abstract

Larvae of black soldier flies, Hermetia illucens, are increasingly used for biological conversion of animal and plant wastes into ingredients of animal feeds on an industrial scale. The presence of pathogenic microorganisms in harvested larvae may be a serious problem for wide-scale adoption of this technology. Fortunately, black soldier fly larvae may have some antimicrobial properties. Methicillin-resistant Staphylococcus aureus (MRSA) is a bacterium associated with various environments that can be pathogenic to humans and farmed animals. We tested whether black soldier fly larvae suppress MRSA on potato substrate. Autoclaved potatoes containing black soldier fly larvae (P+BSFL), potatoes inoculated with MRSA and containing black soldier fly larvae (P+MRSA+BSFL), and potatoes inoculated with MRSA (P+MRSA) were incubated in glass jars. Substrate samples were taken after 3 and 7 days of incubation and plated on Trypticase soy agar (TSA) and Staphylococcus medium 110 agar (SA) to quantify total bacteria and MRSA, respectively. DNA was extracted from potato substrates on both days and sequenced to assess bacterial and fungal diversity using 515F/806R and internal transcribed spacer (ITS) 1/2 primers, respectively, and QIIME 2.0 software. Both total bacterial and MRSA-specific CFU were reduced in the presence of black soldier fly larvae, with a larger reduction for the latter. Twenty-five bacterial genera and 3 fungal genera were detected. Twenty bacterial genera were shared among the treatments and the days, but their relative abundances often varied. Among the most abundant genera, only *Enterococcus* and *Lactococcus* were universally present. Our findings confirm antimicrobial properties of black soldier fly larvae.

**IMPORTANCE** Larvae of black soldier flies, Hermetia illucens, may be used to provide an environmentally sustainable and economically viable method for biological conversion of animal and plant wastes into ingredients of animal feeds on an industrial scale. However, contamination of harvested larvae by pathogenic microorganisms inhabiting decaying substrates may be a serious problem for wide-scale adoption of this technology. Fortunately, black soldier fly larvae may have some antimicrobial properties, including suppression of several common pathogens. Our study showed that such a suppression applies to methicillin-resistant Staphylococcus aureus, which is a ubiquitous bacterium pathogenic to animals (including humans).

## INTRODUCTION

Finding alternative feeds in agriculture is important for ensuring sustainability of farming under the conditions of decreasing availability of farmland and climate change (1). Integration of insects into the diets of farmed animals has become an attractive option. Insects are nutritious, more sustainable than other feeds, and widely accepted as feed by several species of domesticated animals. The black soldier fly (Hermetia illucens [BSF]), particularly its larvae (BSFL), has emerged as a popular alternative feed ingredient for several reasons. BSFL are a quick-developing and prolific species that produce 500 to 900 eggs per female (2). These detritivorous insects can develop on numerous substrates, including food waste, fecal waste of humans and other animals, and a variety of organic by-products from food, agriculture, and aquaculture industries (2). Capturing nutrients that are otherwise lost in these wastes is beneficial to the food cycle and the environment, especially taking into consideration the increases in resource scarcity throughout global communities (2).

A common application for industrial BSFL rearing includes bioconversion of waste into larval biomass. The rearing of BSFL on organic waste has become an increasingly common industry for recycling organic waste and generating livestock feed ingredients (2, 3). BSFL have been successfully integrated into the diets of poultry, pigs, fish, and crustaceans during experimental trials (3, 4).

Concerns have been raised regarding the long-term safety and security of BSFL use, particularly in relation to the accumulation of microbes within harvested BSFL biomass and within their substrate (5). It is imperative to ensure that BSFL rearing facilities are not contributing to the proliferation and spread of pathogenic bacteria. Investigations into the relationship between larvae and various pathogens that potentially colonize substrates are necessary to ensure the safety of insects as feed.

Several *in vitro* studies have been conducted on BSFL showing they are capable of dramatic alteration of substrates they inhabit (6, 7), including suppressing pathogenic bacterial growth in a variety of organic wastes (2, 6, 8, 9). In particular, BSFL suppress the growth of many Gram-negative bacterial species, such as Klebsiella pneumoniae, Neisseria gonorrhoeae, Shigella sonnei, Escherichia coli O157:H7, and Salmonella spp. (including Salmonella enterica serovar Enteritidis) (7–11). There are also reports showing their suppressive abilities on Gram-positive lactobacilli, group D streptococci, and *Enterococcus* spp. (10, 12). Evidence indicating BSFL suppression of Gram-positive species at the *in vivo* level is an emerging topic (10, 13).

Methicillin-resistant Staphylococcus aureus (MRSA) is a variant of S. aureus resistant to the antibiotic methicillin (14). While MRSA is of particular concern as a nosocomial pathogen, it is also found ubiquitously in agricultural and slaughterhouse settings (15). MRSA has previously been detected in meat and dairy products (15, 16), in animal housing environments (17), and on the skin of farmed animals (15) and in their fecal matter (18). It is known to cause sickness among farm animals, particularly mastitis in dairy cows (15). Furthermore, one of the most prevalent causes of food poisoning in both animals and humans is the ingestion of enterotoxins produced by staphylococcal species (19, 20). Enterotoxigenic strains of S. aureus have been detected in multiple foods, including, but not limited to, eggs, dairy, poultry, and potato products (20). Contamination of meat and dairy products by MRSA strains has also been linked to foodborne infections of humans (21, 22). Staphylococcal enterotoxins can survive pasteurization, radiation, and heat treatments (20). Hospitalization may be necessary for elderly, immunocompromised, or infant patients suffering from staphylococcal food poisoning (20, 23).

Previous research has shown a reduction in the populations of several strains of S. aureus over time in organic substrates inhabited by BSFL (6, 13). However, to the best of our knowledge, no information is currently available on this topic regarding MRSA, despite its importance to public health (13). The goal of our study was to investigate changes in bacterial community in response to the presence of BSFL within contaminated potato substrate, with a particular reference to MRSA colonization.

Disposal of unmarketable cull potato tubers is often a problem for farmers because there are limited opportunities for their processing. When left as unattended piles on farms, these tubers serve as a source of inoculum for several important potato diseases (24). Our earlier study showed that such tubers are a suitable substrate for BSFL growth and development (25). However, potato is known to support growth of S. aureus (20). Therefore, we focused this investigation on simulated potato waste.

## RESULTS

### Total bacterial counts.

Treatments had a significant effect on total bacterial counts (df = 2, 30; *F* = 10.85; *P* = 0.0003). However, there was also a significant difference among the trials (df = 2, 30; *F* = 7.85; *P* = 0.0018). The first trial that was conducted without Trypticase soy broth (TSB) in the pathogen-free treatment was statistically different from the second trial (Tukey’s test, *t* = −2.84, *P* = 0.0213) and the third trial (Tukey’s test, *t* = −3.72, *P* = 0.0023), both of which were conducted with supplemental TSB in that treatment. At the same time, the latter two trials were not different from each other (Tukey’s test, *t* = −1.23, *P* = 0.4464). Therefore, we also analyzed results of the trials conducted with and without TSB separately.

**FIG 1:**
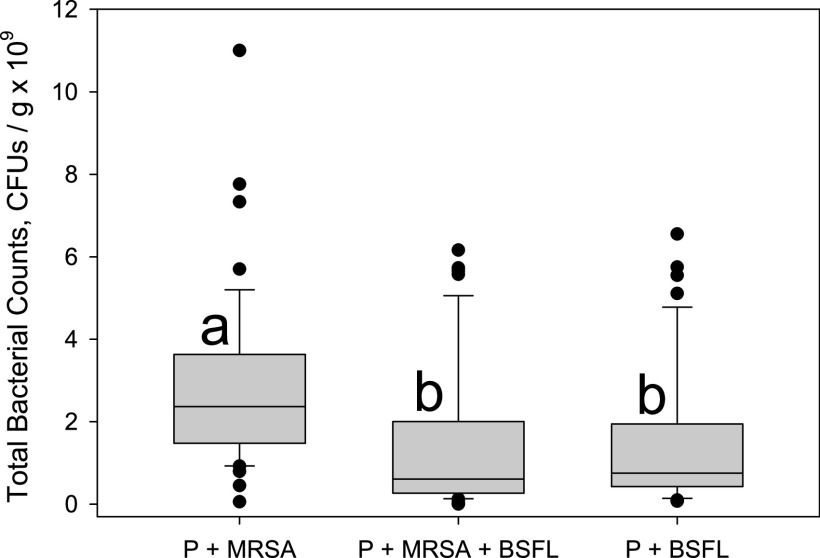
Box plots of total bacterial counts recorded in samples from three different substrates in the second and third trials. P+MRSA denotes potato substrate inoculated with MRSA, P+MRSA+BSFL denotes potato substrate inoculated with MRSA and containing black soldier fly larvae, and P+BSFL denotes uninoculated potato substrate containing black soldier fly larvae. Trypticase soy broth was added to the P+BSFL treatment. Substrate samples were taken after 3 and 7 days of incubation in glass jars at room temperature and plated on Trypticase soy agar (TSA) and Staphylococcus medium 110 agar (SA) to quantify total bacteria and MRSA, respectively. Data for both sampling days were pooled in the absence of a significant treatment-by-day interaction. Overall treatment effect was significant (*P* = 0.0002). Boxes followed by the same letter were not different from each other (Tukey’s test *P* > 0.05).

For the first trial, treatment effects on total bacterial counts were not significant (df = 2, 12; *F* = 2.25; *P* = 0.1474). However, absence of TSB in the uninoculated jars complicated the interpretation of those results. For the pooled results of the second and third trials, treatment effects were significant (df = 2, 21, *F* = 13.67; *P* = 0.0002), while effects of the sampling day (df = 1, 21; *F* = 0.88, *P* = 0.3581) and the interactions of the two factors (df = 2, 21; *F* = 2.49; *P* = 0.1072) were not significant. Both treatments containing black soldier fly larvae (potato plus MRSA plus BSFL [P+MRSA+BSFL] and potato plus BSFL [P+BSFL]) had significantly reduced total bacterial counts in their substrates compared to the treatment containing only potato inoculated with MRSA (P+MRSA) but were not different from each other (Fig. 1).

### MRSA counts.

**FIG 2:**
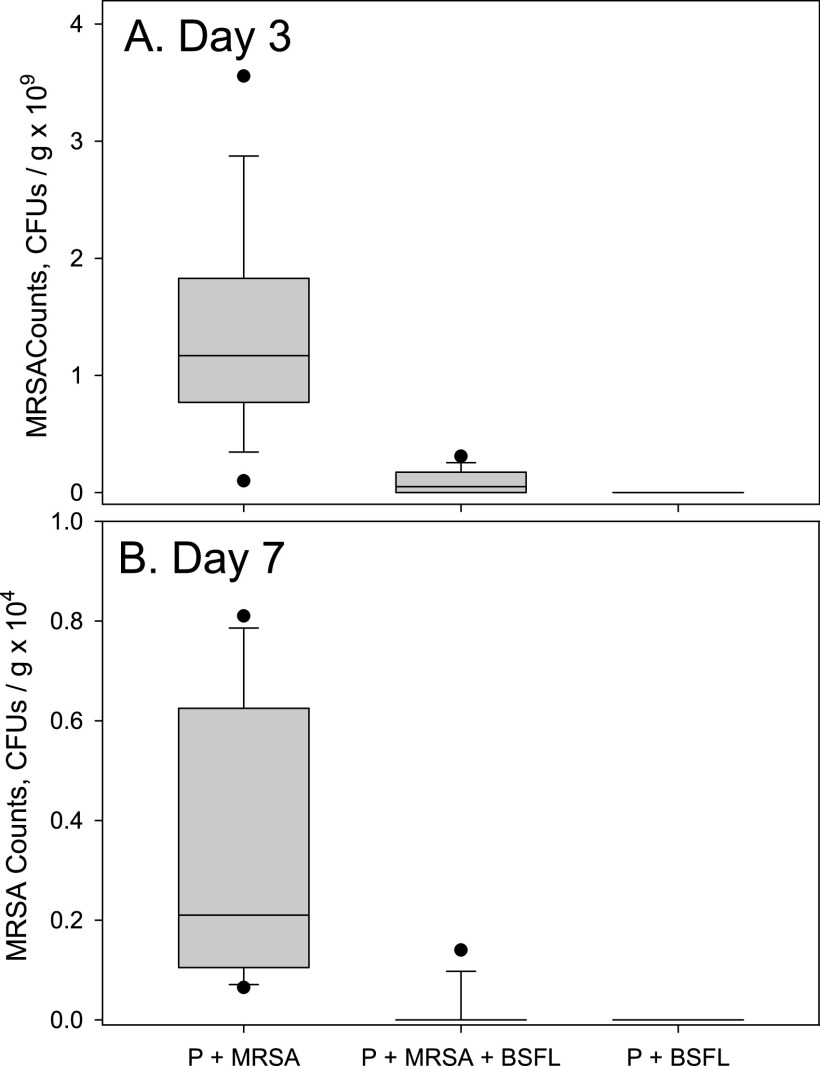
Box plots of MRSA counts recorded in samples from three different substrates in the first trial. Please note the difference in scales between panels A and B. P+MRSA denotes potato substrate inoculated with MRSA, P+MRSA+BSFL denotes potato substrate inoculated with MRSA and containing black soldier fly larvae, and P+BSFL denotes uninoculated potato substrate containing black soldier fly larvae. Substrate samples were taken after 3 and 7 days of incubation in glass jars at room temperature and plated on Trypticase soy agar (TSA) and Staphylococcus medium 110 agar (SA) to quantify total bacteria and MRSA, respectively. No Trypticase soy broth (TSB) was added to the P+BSFL treatment. Treatment effects were significant on both days of sampling (*P* < 0.0001).

In the first trial, significant differences were detected both among the treatments (df = 2, 12; *F* = 134.46; *P* < 0.0001) as well as between the days of sampling (df = 1, 12; *F* = 47.98; *P* < 0.0001). The interaction between these two variables was also significant (df = 2, 12; *F* = 12.07; *P* = 0.0013). Fewer MRSA cells were counted in samples taken on day 7 than in samples taken on day 3 (Fig. 2). On both days, none of the uninoculated samples (P+BSFL) had detectable levels of MRSA (below the 10^7^-CFU/g plating threshold) (Fig. 2). Inoculated samples (P+MRSA and P+MRSA+BSFL) had significantly fewer MRSA counts when black soldier fly larvae were present (Fig. 2) on day 3 (df = 2, 12; *F* = 104.36; *P* < 0.0001) and day 7 (df = 2, 12; *F* = 41.98; *P* < 0.0001). However, the observed difference was larger on day 3 (Fig. 2).

**FIG 3:**
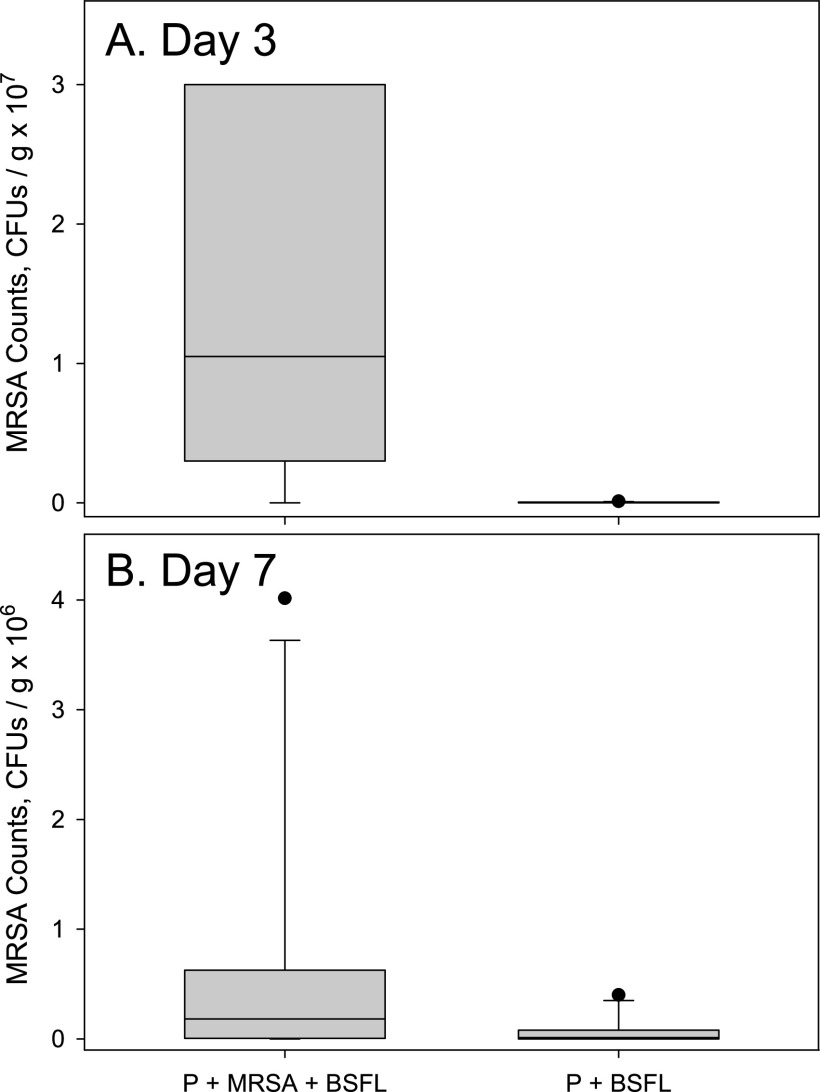
Box plots of MRSA counts recorded in samples from three different treatments in the second trial. Please note the difference in scale between panels A and B. P+MRSA+BSFL denotes potato substrate inoculated with MRSA and containing black soldier fly larvae, and P+BSFL denotes uninoculated potato substrate containing black soldier fly larvae. Substrate samples were taken after 3 and 7 days of incubation in glass jars at room temperature and plated on Trypticase soy agar (TSA) and Staphylococcus medium 110 agar (SA) to quantify total bacteria and MRSA, respectively. Colonies from potato substrate inoculated with MRSA could not be quantified because of confluence. Trypticase soy broth (TSB) was added to the P+BSFL treatment. Treatment effects were significant on both days of sampling (*P* = 0.0013).

In the second trial, staphylococcal media from samples not containing black soldier fly larvae (P+MRSA) were mostly confluent at both dilutions. As a result, quantification of MRSA was not possible, and that treatment was excluded from subsequent analysis. The difference between the other two treatments was significant (df = 1, 8; *F* = 18.40; *P* = 0.0027), while MRSA counts were similar on both sampling days (df = 1, 8; *F* = 2.05; *P* = 0.1903). There was a significant interaction between the treatment and the day of sampling (df = 1, 8; *F* = 5.81; *P* = 0.0424). Inoculated samples (P+MRSA+BSFL) had more staphylococcal colonies on day 3 (Fig. 3A) (df = 1, 8; *F* = 23.54; *P* = 0.0013) but not on day 7 (Fig. 3B) (df = 1, 8; *F* = 2.46; *P* = 0.1554).

**FIG 4:**
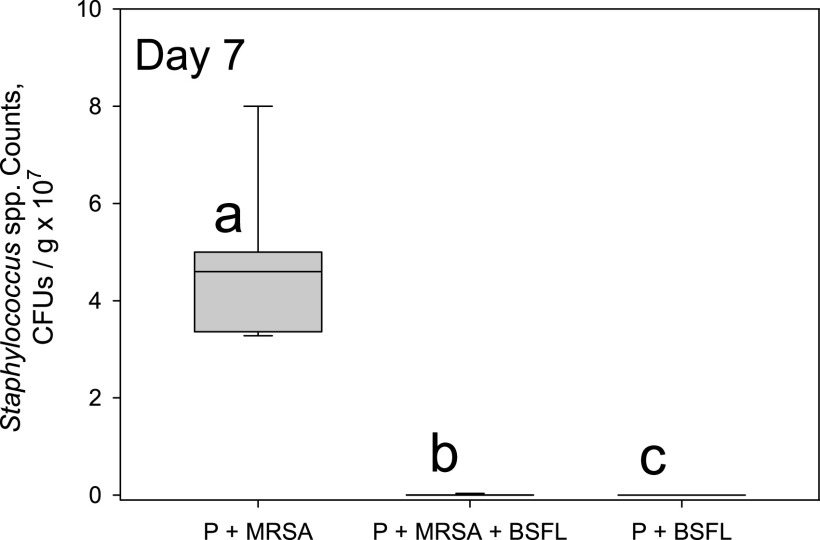
Box plots of MRSA counts recorded in samples from three different substrates in the third trial. P+MRSA+BSFL denotes potato substrate inoculated with MRSA and containing black soldier fly larvae, and P+BSFL denotes uninoculated potato substrate containing black soldier fly larvae. Substrate samples were taken after three and 7 days of incubation in glass jars at room temperature and plated on Trypticase soy agar (TSA) and Staphylococcus medium 110 agar (SA) to quantify total bacteria and MRSA, respectively. Trypticase soy broth (TSB) was added to the P+BSFL treatment. Overall treatment effect was significant (*P* < 0.0001). Boxes followed by the same letter were not different from each other (Tukey’s test *P* > 0.05). No data are presented for day 3 because colonies in both inoculated treatments became confluent.

In the third trial, overgrowth on selective media in both inoculated treatments (P+MRSA and P+MRSA+BSFL) on day 3 prevented enumeration. Occasional colonies were present on uninoculated plates, but at densities not exceeding 1 to 2 CFU. Therefore, inclusion of those readings for statistical analyses was not possible. On day 7, there was a significant difference among the treatments (df = 2,23; *F* = 58.93; *P* < 0.0001). Similar to the first trial, uninoculated samples (P+BSFL) had no detectable staphylococci (below the 10^4^-CFU/g plating threshold), while the presence of black soldier fly larvae significantly reduced the number of staphylococcal colonies present in substrate (Fig. 4).

### Microbial community composition.

Analysis of the potato substrate using Illumina MiSeq technology showed the presence of 25 unique bacterial genera and 3 fungal genera (Table 1). Bacterial populations were different by treatment and day; however, fungal reads were overwhelmingly *Trichosporon* spp. (Trichosporonales: Trichosporonaceae) in all samples. *Corynebacterium*, *Enterococcus*, *Lactococcus*, and *Weissella* were found to make up a large proportion of the bacterial community. In contrast, *Clostridium* and *Bacillus* were substantially less prominent. The jars containing BSFL and MRSA (P+MRSA+BSFL) had *Corynebacterium* (day 3) and *Weissella* (day 7) as the most prevalent genera in the substrate. These results differed for the treatment without MRSA (P+MRSA+BSFL), where the largest numbers of reads were *Lactococcus* on day 3 and *Enterococcus* on day 7 of sampling.

**TABLE 1:** Genera of bacteria and fungi detected through 16S rRNA gene and ITS sequencing, respectively, in the samples of potato substrates inhabited by black soldier fly larvae for 3 or 7 days

Genus	Bacterial or fungal genus detected on substrate^*a*^							
	P+MRSA+BSFL				P+BSFL			
	Day 3		Day 7		Day 3		Day 7	
	Counts	Relative %	Counts	Relative %	Counts	Relative %	Counts	Relative %
Bacteria								
*Actinomyces*	62	0.19	366	1.09	129	0.41	226	0.68
*Azospira*	9	0.03	216	0.64	26	0.08	62	0.19
*Bacillus*	681	2.12	81	0.24	62	0.20	359	1.08
*Campylobacter*	43	0.13	0	0.00	114	0.36	1	0.00
*Clostridium*	34	0.11	1,407	4.20	465	1.47	574	1.72
*Corynebacterium*	11,477	35.76	78	0.23	293	0.93	11,552	34.64
*Dysgonomonas*	133	0.41	821	2.45	262	0.83	1,483	4.45
*Enterococcus*	6,471	20.16	4,721	14.09	4,697	14.83	13,649	40.93
*Erysipelothrix*	93	0.29	0	0.00	4	0.01	9	0.03
*Ignatzschineria*	3	0.01	11	0.03	8	0.03	5	0.01
*Klebsiella*	469	1.46	64	0.19	55	0.17	103	0.31
*Lachnoclostridium*	1	0.00	29	0.09	5	0.02	13	0.04
*Lactococcus*	5,849	18.23	4,930	14.71	10,298	32.52	2,178	6.53
*Lactonifactor*	57	0.18	72	0.21	32	0.10	59	0.18
*Morganella*	3,898	12.15	246	0.73	1,382	4.36	886	2.66
*Paenibacillus*	14	0.04	1	0.00	0	0.00	57	0.17
*Porphyromonas*	12	0.04	68	0.20	16	0.05	18	0.05
*Proteus*	49	0.15	0	0.00	1	0.00	9	0.03
*Providencia*	767	2.39	95	0.28	242	0.76	145	0.43
*Raoultella*	18	0.06	0	0.00	0	0.00	2	0.01
*Salana*	65	0.20	2	0.01	281	0.89	8	0.02
*Staphylococcus*	38	0.12	452	1.35	7,885	24.90	58	0.17
*Trabulsiella*	820	2.56	13	0.04	159	0.50	40	0.12
*Vagococcus*	948	2.95	32	0.10	267	0.84	36	0.11
*Weissella*	81	0.25	19,806	59.10	4,987	15.75	1,814	5.44
Fungi								
*Aspergillus*	5	0.01	76	0.22	76	0.23	2	0.01
*Cladosporium*	0	0.00	1	0.01	1	0.00	2	0.01
*Trichosporon*	33,613	99.99	33,353	99.77	33,353	99.77	33,497	99.80

a P+MRSA+BSFL denotes potato substrate inoculated with MRSA and containing black soldier fly larvae, and P + BSFL denotes uninoculated potato substrate containing black soldier fly larvae.

Of the 25 bacterial genera, 20 were shared among the treatments and the days. The remaining five were not specific to one treatment but were shared among two or three treatments. Those genera were, as a whole, seldomly encountered in our study (Table 1). Relative abundances of the shared genera often varied between different treatments and days. When we compared the genera comprised by more than 1% of the mapped reads in each treatment-day combination, only *Enterococcus* and *Lactococcus* were universally present (Table 2). Interestingly, *Weissella* was almost completely absent from the substrate inoculated by MRSA after 3 days of incubation but dominated the microbial community in the same treatment after 7 days (Table 1).

**TABLE 2:** Overlap in the common genera (>1% of mapped reads) of bacteria detected through 16S rRNA gene sequencing of the samples of potato substrates inhabited by black soldier fly larvae for 3 or 7 days

Genus	Overlap in genera on substrate^*a*^			
	P+MRSA+BSFL		P+BSFL	
	Day 3	Day 7	Day 3	Day 7
*Actinomyces*		x		
*Bacillus*	x			x
*Clostridium*		x	x	x
*Corynebacterium*	x			x
*Dysgonomonas*		x		x
*Enterococcus*	x	x	x	x
Klebsiella	x			
*Lactococcus*	x	x	x	x
*Morganella*	x		x	x
*Providencia*	x			
Staphylococcus		x	x	
*Trabulsiella*	x			
*Vagococcus*	x			
*Weissella*		x	x	x

a P+MRSA denotes potato substrate inoculated with MRSA, P+MRSA+BSFL denotes potato substrate inoculated with MRSA and containing black soldier fly larvae, and P+BSFL denotes uninoculated potato substrate containing black soldier fly larvae.

### Bacterial diversity index.

Data compiled from reads mapped to operational taxonomic units (OTUs) indicated bacterial diversity was lowest in potato spiked with MRSA and fed on by BSFL (P+MRSA+BSFL) on day 7 (Table 3). All remaining samples were similar in terms of bacterial diversity expressed by the Shannon-Wiener diversity index. Simpson’s diversity index was 0.99 for all samples, indicating heavy dominance by a few genera. Sequence sampling depth did not impact diversity indices following rarefaction (data not shown).

**TABLE 3:** Bacterial community characteristics based on 16S rRNA gene sequencing data on days 3 and 7 in potato substrate fed on by black soldier fly larvae with and without MRSA

Day	Treatment	Mapped reads (*n*)^*a*^	Genera (*n*)	Shannon-Wiener index value (H′)
3	P+MRSA +BSFL	32,092	25	1.83
	P+BSFL	31,670	23	1.80

7	P+MRSA+BSFL	33,511	21	1.37
	P+BSFL	33,346	25	1.80

a Total number of reads assigned to an operational taxonomic unit.

## DISCUSSION

Our results confirmed antibacterial properties of BSFL. Both total bacterial and MRSA populations were significantly reduced in their presence. This is consistent with other research published over the past 2 decades. Suppressive capabilities of BSFL were originally shown against Gram-negative bacterial species (2, 8, 9). Whole larval biomass has suppressed Klebsiella pneumoniae, Neisseria gonorrhoeae, and Shigella sonnei, and larval extracts have inhibited Escherichia coli and Pseudomonas fluorescens (26). *In vivo*, BSFL suppressed Salmonella in human feces and Escherichia coli among chicken and dairy manures (10, 27). Furthermore, BSFL were capable of suppressing *Bacteroides* and *Proteobacteria* in unsterilized chicken manure after 15 days (11).

As with Gram-negative bacteria, studies have shown BSFL suppression of Gram-positive bacteria. Species such as Clostridium perfringens, Micrococcus luteus, and Bacillus subtilis, as well as those of the lactobacilli and group D streptococci, have been suppressed *in vitro* by larval extracts (12, 26, 28). Analysis of some larval extracts by Dong et al. (28) found lauric acid and medium-chain fatty acid derivatives in high concentrations with antimicrobial effects against Gram-positive bacteria (28). Additionally, factors like pH and the enzymatic reactions of the BSFL gut are believed to reduce bacterial populations (29, 30). Characterization of antimicrobial peptides, such as the defensin-like peptides, found in BSFL have proven antibacterial effects on both Gram-positive and Gram-negative bacteria (29, 31, 32). Specifically, defensin-like peptide 4 (DLP4) showed antibacterial activity against MRSA (32).

*In vivo* research also supports the suppressive capabilities of BSFL toward Gram-positive bacteria in various organic substrates (2, 10, 11, 13). *Enterococcus* populations in human feces were lowered due to the presence of BSFL (10). Staphylococcus aureus counts from organic waste streams and chicken feed were reduced in the presence of BSFL as well (13). Results obtained from this project indicate that methicillin-resistant S. aureus should be added to the list of Gram-positive bacteria suppressed by BSFL.

In the present study, black soldier fly larvae were not subjected to full sterilization prior to introduction to sterile potato substrates. Larval excrement and bacteria residing in larval alimentary canals are believed to have contributed to the presence of bacteria in the potato substrates containing BSFL based on the results of several other studies (33, 34). For example, *Enterococcus*, *Dysgonomonas*, *Morganella*, and Staphylococcus, which were found in potato substrate in our study, were also present in the intestinal tract of BSFL, particularly within midgut and hindgut regions (33). The fate of the bacteria that were not observable in the BSFL-treated substrates in our study is unknown. They could have been destroyed entirely by digestion; alternatively, the larvae could have become their reservoir and potential vectors. Further investigation of this issue is needed and may be particularly informative for the use of BSFL as a component of a bioremediation strategy.

Treatments not inoculated with MRSA did not contain detectable levels of MRSA colonies. Inoculated treatments had significantly fewer CFU on selective media in the presence of BSFL, suggesting that BSFL have a suppressive effect on MRSA. Gorrens et al. (13) found that chicken feed artificially inoculated with S. aureus had lower bacterial counts in BSFL-containing treatments, indicating some level of antimicrobial activity is present (13). Similar results from Huang et al. (35) found inhibition of S. aureus in pig manure within treatments containing BSFL. Bacterial counts dropped 3-fold after 8 days in BSFL treatments and increased 1-fold in treatments lacking BSFL (35). Initial suppression of both Gram-negative and Gram-positive species has been hypothesized to occur as a result of antimicrobial peptide production within larval hemolymph, resulting in cell membrane disruption and subsequent microbial death (2). Recent evidence has suggested peptides such as defensins are capable of selective inhibition of Gram-positive species, yet evidence confirming this phenomenon is minimal (36, 37).

The biological processes that contribute to the reduction of MRSA within the potato substrate are hypothesized to originate from either competitive inhibition with the dominant microflora or exposure to antimicrobial components of BSFL origin. The antimicrobial properties of BSFL biomass and their accompanying microflora have been investigated, with recent reports suggesting synergistic microflora-protein interaction effects that contribute to overall reduction of noncommensal bacteria within larval biomass (13, 35). These synergistic interactions with pathogenic bacteria include direct exposure to larval peptides within the midgut regions or prolonged exposure to microbial peptides produced commensally (35). No inhibition assays were conducted with larval microflora isolates within this investigation, yet previous studies have reported increased reduction of S. aureus colonies in the presence of *Trichosporon* spp (13).

Potato tubers are composed of long-chain carbohydrates contained in an aerobic environment, a combination of which can contribute to an increased glucose concentration due to degradation of these starch chains over prolonged periods (38, 39). This is expected to benefit fast-growing fermenters like *Enterococcus* and *Lactococcus*, which dominated microbial communities in this study. Bacteria that are pathogenic to either plant or mammalian systems are likely to compete for substrate with the surrounding microflora, which may contribute to the reduction of such species as S. aureus (40).

The diversity of bacteria surviving in the jars with BSFL was low. Mapped reads obtained from 16S rRNA gene sequencing of extracted DNA were similar across treatments and days (Table 1). Similar numbers of the same bacterial genera were found among treatments and days. Given the artificial introduction of one bacterium (MRSA) into sterilized potato substrate, it could be expected that bacterial diversity would not be high. However, the introduction of BSFL allowed for additional microflora and could be tied to the high diversity observed in other studies (33, 34).

As a result of 16S rRNA gene and fungal internal transcribed spacer (ITS) sequencing, 25 bacterial genera and three fungal genera were detected. The three bacterial genera dominating the total bacterial community were *Enterococcus*, *Lactococcus*, and *Weissella*. While fungi were not our focus, *Trichosporon* populations were consistently the highest among all treatments and days (data not shown). There is evidence that *Trichosporon* plays a large role in the microbiota of BSFL (41). In addition, the presence of this genus within the commensal microbiota could contribute to decreased counts of Staphylococcus aureus (13).

Consistent relationships between the presence of Staphylococcus species and other bacteria were not easily observed (Table 1 and Table 2). The high prevalence of *Weissella* and low prevalence of Staphylococcus on day 7 of the BSFL with pathogen treatment could be attributed to their previously documented competitive relationship (42). However, this is not supported by the results from the BSFL with potato treatment. All samples showed a higher presence of *Enterococcus* or *Lactococcus* over Staphylococcus spp. Research has shown that when cocultured with *Enterococcus* or *Lactococcus*, staphylococcal growth is reduced (42, 43).

An interesting result of this study is the observance of fewer Staphylococcus reads in the 16S sequencing data than might be expected, especially in the treatments that were inoculated with MRSA. One explanation for these results is that total bacterial counts were >10^9^ CFU/g for all samples, but MRSA-specific counts were on the order of 10^4^ CFU/g (a difference of 5 orders of magnitude) by day 7. With a read depth of only ~30,000 (Table 3), we might expect that staphylococcal reads in each treatment would be fewer if MRSA is the major species of Staphylococcus present. Since total staphylococci were not enumerated in this study (only colonies consistent with MRSA were counted), the full staphylococcal burden within the substrate could not be determined. It would be useful to enumerate total staphylococci in future studies to help answer this question.

Beyond having documented interactions with Staphylococcus spp., many of the bacterial genera with relative percentages higher than 1% also have a known relationship with BSFL. Several studies have compared the gut microbiome of BSFL and found that the bacterial makeup is significantly influenced by diet (2, 34). However, there has been a consensus that regardless of external influences, the common taxonomic classes of microflora include, but are not limited to *Bacilli*, *Gammaproteobacteria*, and *Bacteroidia* (44). More recent work has found additional commensal genera, such as *Enterococcus*, *Dysgonomonas*, *Providencia*, *Morganella*, and Staphylococcus, in the gut microbiome of BSF larvae (33, 45). Samples taken from BSFL-containing vermicompost had a higher abundance of *Corynebacterium*, *Enterococcus*, *Vagococcus*, and *Providencia* than those lacking BSFL (46). *Corynebacterium* was the dominant genus among BSFL-containing samples (46). The results in Table 1 include these classes and genera of bacteria, potentially linking the BSFL excreta in the substrate to the diversity of bacteria detected through purification and sequencing. The top three bacterial genera found across all treatments and days are Gram-positive. Few Gram-negative bacteria were present in sampled substrates.

The processing of organic waste using black soldier fly larvae is a novel bioremediation method that has potential uses in large-scale industrial food and agricultural feed applications. The suppressive effects of BSFL on the clinically important MRSA isolates are likely to contribute to the safety of feed ingredients and composts produced in the process of recycling potato wastes. Overall reduction in MRSA colonies was sustained within potato substrate throughout the exposure period, suggesting larval excretions may have bacteriostatic capabilities due to digestive enzymes, antimicrobial peptides, and/or competitive microorganisms. Further investigations will include characterization of antimicrobial contents present and identification of bacterial species that participate in preserving microbiome integrity in larval flora.

## MATERIALS AND METHODS

### Sterilization and preparation of materials.

Several 1-pt (473-mL) glass jars, metal lid screw bands, and cheesecloth were sterilized by autoclaving (121°C, 18 lb/in^2^, 30 min). Organic russet potatoes (Morning Kiss Organic, Chelsea, MA) were purchased in 3-lb quantities from the local supermarket for use as the substrate. These potatoes were rinsed with deionized water and sterilized by autoclaving (121°C, 18 lb/in^2^, 30 min). Following sterilization, potatoes were cooled for 30 to 45 min and manually homogenized with a sterile Scoopula. Processed potatoes were weighed, and 165 g was placed into presterilized 473-mL Mason jars. Five random jars were sampled with a sterile cotton swab and streaked onto Trypticase soy agar (TSA) (BD Biosciences, Franklin Lakes, NJ) plates. Growth was assessed after 24 h in an incubator at 37°C with 95% relative humidity to ensure a sterile substrate was achieved.

### Establishing treatment groups.

Three experimental treatment groups were created: potato with methicillin-resistant Staphylococcus aureus (P+MRSA), potato with black soldier fly larvae (P+BSFL), and potato with BSFL and MRSA (P+BSFL+MRSA). A laboratory-maintained strain of MRSA was inoculated into 50 mL of Trypticase soy broth (TSB) (BD Biosciences, Franklin Lakes, NJ) and incubated for 18 to 20 h at 37°C with 95% relative humidity. To inoculate the potato substrates, this culture was diluted to an optical density at 600 nm (*A*_600_) of 0.3 (measured by dilution plating as 10^8^ CFU/mL). This dilution was added to appropriate treatment substrates at a rate of 0.01 mL/g culture based on the relationship of volume (0.01 mL) per substrate weight (grams) previously utilized (27).

BSFL used in these experiments were purchased from Symton BSF Company (College Station, TX), where they are maintained on Gainesville diet (50% wheat bran, 30% alfalfa, and 20% cornmeal [vol/vol]) (47, 48). Upon receipt, the colony was maintained in 8-qt ventilated plastic containers at 25°C. Larvae were fed unsterilized potatoes *ad libitum* for 48 h to minimize the effects of dietary changes during bioassays. Preparation of the larvae for experiments began with physical extraction of larvae from the substrate using featherweight forceps. All experimental larvae were between the second and third developmental instar stages. Larvae were rinsed with 700 mL of distilled water to remove substrate traces before being introduced into experimental substrates. A total of 50 larvae were introduced into each jar.

Three trials were conducted in total. The first two trials had five jars per treatment. The third trial had three jars per treatment. In the second and third trials, the non-MRSA treatments also had a 0.01-mL/g culture of TSB (27). This was completed to account for any possible stimulating effect on bacterial growth in jars.

### Sampling and plating of substrate.

Larvae were prepared for bioassays by air drying them for a period of 30 min on top of dry paper towels. Further sterilization was not done to avoid damaging the larvae or creating an environment that would not represent *in vivo* studies. Larval batches were weighed and counted following air drying. At the beginning of the experiments, an average larva weighed ca. (6.8 ± 1.2) × 10^−3^ g (mean ± standard deviation [SD]).

After the components necessary for each treatment group were added, each jar was covered in sterile cheesecloth and secured using a sterile metal screw band. Jars were stored in an empty chemical fume hood with the sash lowered and at ambient temperature under natural daylight.

Three substrate samples were taken from each jar on days 3 and 7 of the experiment. To collect samples, substrates were homogenized within the jars and 5 g was removed, aseptically. Care was exercised during collection to avoid damage or disturbance to larvae. Each 5-g sample was added to 45 mL of sterile water and gently swirled (50 to 100 rpm) for 5 to 10 min using a benchtop orbital shaker. The triplicate batches of flasks were then serially diluted and plated onto both TSA and *Staphylococcus* medium 110 (BD Difco, BD Biosciences, Franklin Lakes, NJ) agar (SA) for enumeration of total bacteria and staphylococci, respectively. The average CFU per gram of total bacteria were collected from TSA (an average of dilutions of 10^−7^ and 10^−8^), while the average CFU per gram of MRSA were determined from Staphylococcus medium 110 (an average of dilutions of 10^−4^ and 10^−5^). All bacterial plates were incubated for 24 h at 37°C. Colony counts were recorded as the number of CFU per gram of substrate. Colonies consistent with morphology and reaction of MRSA on staphylococcal 110 medium were enumerated as MRSA.

### Diversity of bacteria surviving in BSFL-inhabited substrates.

Since the potato substrate was sterilized prior to experimentation, it was almost exclusively comprised of the added pathogen, MRSA. That was evidenced by the colonies observed on both selective and nonselective media throughout all trials. Therefore, samples from P+MRSA treatments were not sequenced for microbial community analysis. For the remaining two treatments of the first trial, an additional 1 g of potato was taken from each jar on each day as described above. Samples were pooled by day and treatment type and stored in a plastic centrifuge tube. Each treatment had its own set of tubes, and treatments were not pooled. The tubes were frozen at −20°C until processing. Samples were thawed and subjected to DNA purification and extraction using the protocol outlined in the DNeasy PowerSoil Pro kit (Qiagen, Germantown, MD). Variability in DNA quality was present among the three trials. Extractions from the first trial contained the highest quality DNA; thus, we decided to proceed with the DNA isolates from that trial only. Samples were sequenced by Molecular Research DNA (MR DNA, Shallowater, TX) using their MiSeq protocol. ITS 1/2 primers and 515F/806R were used to assay fungal and bacterial diversity, respectively. Chimeric and singleton sequences were removed, and sequences were denoised and quality filtered according to MR DNA’s proprietary protocol. Sequences were classified into OTUs through a database, including NCBI, GreenGenes, and RDPII (MR DNA, Shallowater, TX).

Diversity was analyzed using QIIME 2.0 (49). Raw bacterial sequencing data were explored using QIIME 2.0 (49) and the recommended workflow for paired-end sequences. Those sequences were processed with Cutadapt (50) scripts to remove primers and adapters and then denoised with Deblur (51). Taxonomic assignments were made using GreenGenes (52) release 13.8. The results of QIIME 2 analysis of bacterial data closely matched those provided by MR DNA (Shallowater, TX), although a few reads failed to map to the genus level.

The NCBI-containing database used by MR DNA is generally thought to be more suitable for assessing biological diversity than GreenGenes alone (53). Therefore, taxonomic assignments provided by MR DNA were used for assessment of diversity characteristics and major and rare taxa associated with the treatments tested in the present study. Diversity characteristics presented herein were calculated from the total number of reads mapped to the species level. Subsequently, species richness (as number of observed different species), the Shannon-Wiener diversity index (54), and Simpson’s diversity index (55) were all calculated from these data.

### Statistical analysis.

The averages between both dilutions of colony counts from TSA plates were standardized to 10^7^. Similarly, the averages between both dilutions of colony counts from *Staphylococcus* medium were standardized to 10^4^. Any counts that were two or more standard deviations from the mean were considered outliers and excluded from analysis.

Data normality was tested using the Kolmogorov-Smirnov test (PROC UNIVARIATE) (56) and found to be nonnormal (*P* < 0.01). Consequently, it was transformed using log transformations, which normalized total bacterial counts on TSA plates but not Staphylococcus counts on SA plates. Therefore, the latter were rank transformed as described by Conover and Iman (57). For ease of viewing, nontransformed data are presented.

The number of total bacterial CFU was analyzed using repeated-measures analysis of variance (ANOVA) (PROC MIXED) (56) with trial, treatment, and day of sampling as the main factors. However, as described below, there was a significant difference between the first trial (TSB added) and the subsequent two trials (no TSB added). Therefore, the three trials were subsequently analyzed separately using repeated-measures ANOVA (PROC MIXED) (56) with treatment and day of sampling as the main factors.

Colonies of Staphylococcus spp. tended to become confluent in the absence of BSFL, making some of the counts too numerous to quantify. Therefore, a global statistical model that included the trial as one of the main factors became seriously unbalanced. As a result, data were analyzed separately for each of the three trials. For the first two trials, repeated-measures ANOVA was used as described above. For the third trial, one-way ANOVA was used (PROC GLM) (56).

When overall treatment effects were significant, they were separated using Tukey’s tests. When treatment-by-day interactions were significant, treatment effects on each of 2 sampling days were tested individually using the SLICE option (PROC MIXED) (56).

## References

[1] Pinotti L, Giromini C, Ottoboni M, Tretola M, Marchis D. 2019. Review: insects and former foodstuffs for upgrading food waste biomasses/streams to feed ingredients for farm animals. Animal 13:1365–1375. doi:10.1017/S1751731118003622. 30691544

[2] Smet JD, Wynants E, Cos P, Van Campenhout L. 2018. Microbial community dynamics during rearing of black soldier fly larvae (*Hermetia illucens*) and impact on exploitation potential. Appl Environ Microbiol 84:e02722-17. doi:10.1128/AEM.02722-17. 29475866 PMC5930328

[3] All About Feed. 2016. EU agrees on insect protein for aquafeed. http://www.allaboutfeed.net/New-Proteins/Articles/2016/12/EU-agrees-oninsect-protein-for-aquafeed-70425E/.

[4] Makkar HPS, Tran G, Heuzé V, Ankers P. 2014. State-of-the-art on use of insects as animal feed. Anim Feed Sci Technol 197:1–33. doi:10.1016/j.anifeedsci.2014.07.008.

[5] Garofalo C, Milanović V, Cardinali F, Aquilanti L, Clementi F, Osimani A. 2019. Current knowledge on the microbiota of edible insects intended for human consumption: a state-of-the-art review. Food Res Int 125:108527. doi:10.1016/j.foodres.2019.108527. 31554102

[6] Awasthi MK, Liu T, Awasthi SK, Duan Y, Pandey A, Zhang Z. 2020. Manure pretreatments with black soldier fly *Hermetia illucens* L. (Diptera: Stratiomyidae): a study to reduce pathogen content. Sci Total Environ 737:139842. doi:10.1016/j.scitotenv.2020.139842. 32526587

[7] Bernard E, Villazana J, Alyokhin A, Rose J. 2020. Colonization of finfish substrate inhabited by black soldier fly larvae by blow flies, bacteria, and fungi. J Insects Food Feed 6:291–304. doi:10.3920/JIFF2019.0044.

[8] Lalander CH, Fidjeland J, Diener S, Eriksson S, Vinnerås B. 2015. High waste-to-biomass conversion and efficient *Salmonella* spp. reduction using black soldier fly for waste recycling. Agron Sustain Dev 35:261–271. doi:10.1007/s13593-014-0235-4.

[9] Choi W-H, Yun J-H, Chu J-P, Chu K-B. 2012. Antibacterial effect of extracts of *Hermetia illucens* (Diptera: Stratiomyidae) larvae against Gram-negative bacteria: *Hermetia illucens* antibacterial activity. Entomol Res 42:219–226. doi:10.1111/j.1748-5967.2012.00465.x.

[10] Lalander C, Diener S, Magri ME, Zurbrügg C, Lindström A, Vinnerås B. 2013. Faecal sludge management with the larvae of the black soldier fly (*Hermetia illucens*)—from a hygiene aspect. Sci Total Environ 458-460:312–318. doi:10.1016/j.scitotenv.2013.04.033. 23669577

[11] Zhang X, Zhang J, Jiang L, Yu Z, Zhu H, Zhang J, Feng Z, Zhang X, Chen G, Zhang Z. 2021. Black soldier fly (*Hermetia illucens*) larvae significantly change the microbial community in chicken manure. Curr Microbiol 78:303–315. doi:10.1007/s00284-020-02276-w. 33141316

[12] Spranghers T, Michiels J, Vrancx J, Ovyn A, Eeckhout M, De Clercq P, Smet SD. 2018. Gut antimicrobial effects and nutritional value of black soldier fly (*Hermetia illucens* L.) prepupae for weaned piglets. Anim Feed Sci Technol 235:33–42. doi:10.1016/j.anifeedsci.2017.08.012.

[13] Gorrens E, Looveren NV, Van Moll L, Vandeweyer D, Lachi D, De Smet J, Van Campenhout L. 2021. *Staphylococcus aureus* in substrates for black soldier fly larvae (*Hermetia illucens*) and its dynamics during rearing. Microbiol Spectr 9:e02183-21. doi:10.1128/spectrum.0218321. 34937197 PMC8694120

[14] Fishovitz J, Hermoso JA, Chang M, Mobashery S. 2014. Penicillin-binding protein 2a of methicillin-resistant *Staphylococcus aureus*: resistance by PBP2a in MRSA. IUBMB Life 66:572–577. doi:10.1002/iub.1289. 25044998 PMC4236225

[15] Wendlandt S, Schwarz S, Silley P. 2013. Methicillin-resistant *Staphylococcus aureus*: a food-borne pathogen? Annu Rev Food Sci Technol 4:117–139. doi:10.1146/annurev-food-030212-182653. 23190141

[16] Sergelidis D, Angelidis AS. 2017. Methicillin-resistant *Staphylococcus aureus*: a controversial food-borne pathogen. Lett Appl Microbiol 64:409–418. doi:10.1111/lam.12735. 28304109

[17] Angen Ø, Nielsen MW, Løfstrøm P, Larsen AR, Hendriksen NB. 2021. Airborne spread of methicillin resistant *Staphylococcus aureus* from a swine farm. Front Vet Sci 8:644729. doi:10.3389/fvets.2021.644729. 34150881 PMC8211894

[18] Szabó I, Beck B, Friese A, Fetsch A, Tenhagen B-A, Roesler U. 2012. Colonization kinetics of different methicillin-resistant *Staphylococcus aureus* sequence types in pigs and host susceptibilities. Appl Environ Microbiol 78:541–548. doi:10.1128/AEM.05327-11. 22081568 PMC3255747

[19] Argaw S, Addis M. 2015. A review on staphylococcal food poisoning. Food Sci Qual Manag 40:59–71.

[20] Hennekinne J-A, Buyser M-LD, Dragacci S. 2012. *Staphylococcus aureus* and its food poisoning toxins: characterization and outbreak investigation. FEMS Microbiol Rev 36:815–836. doi:10.1111/j.1574-6976.2011.00311.x. 22091892

[21] Jamali H, Paydar M, Radmehr B, Ismail S, Dadrasnia A. 2015. Prevalence and antimicrobial resistance of *Staphylococcus aureus* isolated from raw milk and dairy products. Food Control 54:383–388. doi:10.1016/j.foodcont.2015.02.013.

[22] Al-Ashmawy MA, Sallam KI, Abd-Elghany SM, Elhadidy M, Tamura T. 2016. Prevalence, molecular characterization, and antimicrobial susceptibility of methicillin-resistant *Staphylococcus aureus* isolated from milk and dairy products. Foodborne Pathog Dis 13:156–162. doi:10.1089/fpd.2015.2038. 26836943

[23] Murray RJ. 2005. Recognition and management of *Staphylococcus aureus* toxin mediated disease. Intern Med J 35:S106–S119. doi:10.1111/j.1444-0903.2005.00984.x. 16271055

[24] Liu C, Liu Y, Chen S. 2005. Effects of nutrient supplements on simultaneous fermentation of nisin and lactic acid from cull potatoes. Appl Biochem Biotechnol 122:475–484. doi:10.1385/ABAB:122:1-3:0475. 15920257

[25] Alyokhin A, Buzza A, Beaulieu J. 2019. Effects of food substrates and moxidectin on development of black soldier fly, *Hermetia illucens*. J Appl Entomol 143:137–143. doi:10.1111/jen.12557.

[26] Vogel H, Müller A, Heckel DG, Gutzeit H, Vilcinskas A. 2018. Nutritional immunology: diversification and diet-dependent expression of antimicrobial peptides in the black soldier fly *Hermetia illucens*. Dev Comp Immunol 78:141–148. doi:10.1016/j.dci.2017.09.008. 28966127

[27] Liu Q, Tomberlin JK, Brady JA, Sanford MR, Yu Z. 2008. Black soldier fly (Diptera: Stratiomyidae) larvae reduce *Escherichia coli* in dairy manure. Environ Entomol 37:1525–1530. doi:10.1603/0046-225x-37.6.1525. 19161696

[28] Dong L, Ariëns RM, America AH, Paul A, Veldkamp T, Mes JJ, Wichers HJ, Govers C. 2021. Clostridium perfringens suppressing activity in black soldier fly protein preparations. LWT 149:111806. doi:10.1016/j.lwt.2021.111806.

[29] De Smet J, Vandeweyer D, Van Mol L, Lachi D, Van Campenhout L. 2021. Dynamics of *Salmonella* inoculated during rearing of black soldier fly larvae (*Hermetia illucens*. Food Res Int 149:110692. doi:10.1016/j.foodres.2021.110692. 34600687 PMC8505792

[30] Bessa LW, Pieterse E, Marais J, Dhanani K, Hoffman LC. 2021. Food safety of consuming black soldier fly (*Hermetia illucens*) larvae: microbial, heavy metal and cross-reactive allergen risks. Foods 10:1934. doi:10.3390/foods10081934. 34441710 PMC8394208

[31] Park S-I, Yoe SM. 2017. Defensin-like peptide3 from black solder fly: identification, characterization, and key amino acids for anti-Gram-negative bacteria. Entomol Res 47:41–47. doi:10.1111/1748-5967.12214.

[32] Park S-I, Kim J-W, Yoe SM. 2015. Purification and characterization of a novel antibacterial peptide from black soldier fly (*Hermetia illucens*) larvae. Dev Comp Immunol 52:98–106. doi:10.1016/j.dci.2015.04.018. 25956195

[33] Zhineng Y, Ying M, Bingjie T, Rouxian Z, Qiang Z. 2021. Intestinal microbiota and functional characteristics of black soldier fly larvae (*Hermetia illucens*). Ann Microbiol 71:13. doi:10.1186/s13213-021-01626-8.

[34] Wynants E, Frooninckx L, Crauwels S, Verreth C, De Smet J, Sandrock C, Wohlfahrt J, Van Schelt J, Depraetere S, Lievens B, Van Miert S, Claes J, Van Campenhout L. 2019. Assessing the microbiota of black soldier fly larvae (*Hermetia illucens*) reared on organic waste streams on four different locations at laboratory and large scale. Microb Ecol 77:913–930. doi:10.1007/s00248-018-1286-x. 30430196

[35] Huang Y, Yu Y, Zhan S, Tomberlin JK, Huang D, Cai M, Zheng L, Yu Z, Zhang J. 2020. Dual oxidase Duox and Toll-like receptor 3 TLR3 in the Toll pathway suppress zoonotic pathogens through regulating the intestinal bacterial community homeostasis in *Hermetia illucens* L. PLoS One 15:e0225873. doi:10.1371/journal.pone.0225873. 32352968 PMC7192390

[36] Bernard E. 2012. Regulation of virulence in *Rhizoctonia solani*: global gene expression profile and impact of soil amendments on rhizoctonia disease of potato. Electron Theses Diss 1834:208.

[37] Shabuer G, Ishida K, Pidot SJ, Roth M, Dahse H-M, Hertweck C. 2015. Plant pathogenic anaerobic bacteria use aromatic polyketides to access aerobic territory. Science 350:670–674. doi:10.1126/science.aac9990. 26542569

[38] Wall MM. 2005. Storage quality and composition of sweetpotato roots after quarantine treatment using low doses of x-ray irradiation. HortScience 40:424–427. doi:10.21273/HORTSCI.40.2.424.

[39] Luo Z, Chen M, Chen T, She P, Wu Y. 2019. Lactic acid produced by glycolysis contributed to *Staphylococcus aureus* aggregation induced by glucose. Curr Microbiol 76:607–612. doi:10.1007/s00284-019-01666-z. 30895345

[40] Amano S-i, Morota T, Kano Y-k, Narita H, Hashidzume T, Yamamoto S, Mizutani K, Sakuda S, Furihata K, Takano-Shiratori H, Takano H, Beppu T, Ueda K. 2010. Promomycin, a polyether promoting antibiotic production in *Streptomyces* spp. J Antibiot (Tokyo) 63:486–491. doi:10.1038/ja.2010.68. 20571515

[41] Boccazzi IV, Ottoboni M, Martin E, Comandatore F, Vallone L, Spranghers T, Eeckhout M, Mereghetti V, Pinotti L, Epis S. 2017. A survey of the mycobiota associated with larvae of the black soldier fly (*Hermetia illucens*) reared for feed production. PLoS One 12:e0182533. doi:10.1371/journal.pone.0182533. 28771577 PMC5542616

[42] Shah N, Patel A, Ambalam P, Holst O, Ljungh A, Prajapati J. 2016. Determination of an antimicrobial activity of *Weissella confusa*, *Lactobacillus fermentum*, and *Lactobacillus plantarum* against clinical pathogenic strains of Escherichia coli and Staphylococcus aureus in co-culture. Ann Microbiol 66:1137–1143. doi:10.1007/s13213-016-1201-y.

[43] Cretenet M, Nouaille S, Thouin J, Rault L, Stenz L, François P, Hennekinne J-A, Piot M, Maillard MB, Fauquant J, Loubière P, Le Loir Y, Even S. 2011. *Staphylococcus aureus* virulence and metabolism are dramatically affected by *Lactococcus lactis* in cheese matrix: *S. aureus* interaction with *L. lactis* in cheese matrix. Environ Microbiol Rep 3:340–351. doi:10.1111/j.1758-2229.2010.00230.x. 23761280

[44] Zheng L, Crippen TL, Singh B, Tarone AM, Dowd S, Yu Z, Wood TK, Tomberlin JK. 2013. A survey of bacterial diversity from successive life stages of black soldier fly (Diptera: Stratiomyidae) by using 16S rDNA pyrosequencing. J Med Entomol 50:647–658. doi:10.1603/me12199. 23802462

[45] Gorrens E, Van Moll L, Frooninckx L, De Smet J, Van Campenhout L. 2021. Isolation and identification of dominant bacteria from black soldier fly larvae (*Hermetia illucens*) envisaging practical applications. Front Microbiol 12:665546. doi:10.3389/fmicb.2021.665546. 34054771 PMC8155639

[46] Jiang CL, Jin WZ, Tao XH, Zhang Q, Zhu J, Feng SY, Xu XH, Li HY, Wang ZH, Zhang ZJ. 2019. Black soldier fly larvae (*Hermetia illucens*) strengthen the metabolic function of food waste biodegradation by gut microbiome. Microb Biotechnol 12:528–543. doi:10.1111/1751-7915.13393. 30884189 PMC6465238

[47] Hogsette JA. 1992. New diets for production of house flies and stable flies (Diptera: Muscidae) in the laboratory. J Econ Entomol 85:2291–2294. doi:10.1093/jee/85.6.2291. 1464690

[48] Sheppard DC, Tomberlin JK, Joyce JA, Kiser BC, Sumner SM. 2002. Rearing methods for the black soldier fly (Diptera: Stratiomyidae). J Med Entomol 39:695–698. doi:10.1603/0022-2585-39.4.695. 12144307

[49] Bolyen E, Rideout JR, Dillon MR, Bokulich NA, Abnet CC, Al-Ghalith GA, Alexander H, Alm EJ, Arumugam M, Asnicar F, Bai Y, Bisanz JE, Bittinger K, Brejnrod A, Brislawn CJ, Brown CT, Callahan BJ, Caraballo-Rodríguez AM, Chase J, Cope EK, Da Silva R, Diener C, Dorrestein PC, Douglas GM, Durall DM, Duvallet C, Edwardson CF, Ernst M, Estaki M, Fouquier J, Gauglitz JM, Gibbons SM, Gibson DL, Gonzalez A, Gorlick K, Guo J, Hillmann B, Holmes S, Holste H, Huttenhower C, Huttley GA, Janssen S, Jarmusch AK, Jiang L, Kaehler BD, Kang KB, Keefe CR, Keim P, Kelley ST, Knights D, et al. 2019. Reproducible, interactive, scalable and extensible microbiome data science using QIIME 2. Nat Biotechnol 37:852–857. doi:10.1038/s41587019-0209-9. 31341288 PMC7015180

[50] Martin M. 2011. Cutadapt removes adapter sequences from high-throughput sequencing reads. EMBnet J 17:10–12. doi:10.14806/ej.17.1.200.

[51] Amir A, McDonald D, Navas-Molina JA, Kopylova E, Morton JT, Zech Xu Z, Kightley EP, Thompson LR, Hyde ER, Gonzalez A, Knight R. 2017. Deblur rapidly resolves single nucleotide community sequence patterns. mSystems 2:e00191-16. doi:10.1128/mSystems.00191-16. 28289731 PMC5340863

[52] McDonald D, Price MN, Goodrich J, Nawrocki EP, DeSantis TZ, Probst A, Andersen GL, Knight R, Hugenholtz P. 2012. An improved GreenGenes taxonomy with explicit ranks for ecological and evolutionary analyses of bacteria and archaea. ISME J 6:610–618. doi:10.1038/ismej.2011.139. 22134646 PMC3280142

[53] Balvočiūtė M, Huson DH. 2017. SILVA, RDP, Greengenes, NCBI and OTT—how do these taxonomies compare? BMC Genomics 18:114. doi:10.1186/s12864-017-3501-4. 28361695 PMC5374703

[54] Shannon CE. 1948. A mathematical theory of communication. Bell Syst Tech J 27:379–423. doi:10.1002/j.1538-7305.1948.tb01338.x.

[55] Simpson EH. 1949. Measurement of diversity. Nature 163:688–688. doi:10.1038/163688a0.

[56] SAS Institute. 2021. SAS version 9.4. SAS Institute, Cary, NC.

[57] Conover WJ, Iman RL. 1981. Rank transformations as a bridge between parametric and nonparametric statistics. Am Stat 35:124–129. doi:10.2307/2683975.

